# Evaluation of ocular and systemic endpoints after radiation of posterior uveal melanoma – A systematic review and meta-analysis

**DOI:** 10.1016/j.heliyon.2024.e36468

**Published:** 2024-08-22

**Authors:** K. Erikson, A. Heidenreich, V. Labunska, R. Beach, F. Cremers, D. Rades, S. Grisanti, A. Katalinic, V. Kakkassery

**Affiliations:** a Department of Ophthalmology, University of Lübeck, Lübeck, Germany; b Institute of Social Medicine and Epidemiology, University of Lübeck, Lübeck, Germany; c Department of Radiotherapy, University of Lübeck, Lübeck, Germany; d Department of Ophthalmology, Klinikum Chemnitz, Chemnitz, Germany

**Keywords:** Uveal melanoma, Choroidal melanoma, Systematic review, Meta-analysis, Radiotherapy

## Abstract

**Background:**

Due to the large number of radiotherapeutic options for treatment of posterior uveal melanoma (UM), advantages of each option regarding important clinical endpoints have yet to be determined. Therefore, objective of this systematic review was to analyze the numerous pro- and retrospective cohort studies focusing on the efficacy of different radiotherapeutic options for UM in adults, considering local tumor control, overall survival, visual acuity, eye preservation, metastasis, radiation side effects and dose rates.

**Methods:**

The Review was performed based on the Cochrane Handbook of Systematic Reviews. The PubMed database was searched for studies published from January 1st, 2000, up to December 31st, 2021. Research, study selection and critical appraisal was performed by two reviewers. The risk of bias assessment was performed through the revised Cochrane risk of bias tools RoB 2 and ROBINS-I. A meta-analysis of proportions was performed using R (R version 4.1.3, library: meta, procedure: metaprop). This systematic review was registered with Prospero (ID CRD42022311758).

**Results:**

Of 4886 studies identified in the database, a total of 20 studies with 4979 participants were included in the qualitative synthesis. Through critical appraisal with ROBINS-I and RoB 2, studies received a ‘moderate’, ‘serious’ or ‘some concerns’ overall risk of bias. Heterogeneity analysis allowed for meta-analysis of proportion of 3 outcome-therapy combinations: local tumor control with I-125 Brachytherapy (proportion: 0.94, CI 95 %: 0.91–0.98), local tumor control with proton therapy (proportion: 0.96, CI 95 %: 0.92–1.00) and eye preservation with I-125 brachytherapy (proportion: 0.91, CI 95 %: 0.88–0.93). This shows local tumor control to be at 94 % with I-125 brachytherapy and at 96 % with proton therapy, as well as an eye preservation rate of 91 % with I-125 brachytherapy.

**Discussion:**

The evaluation of outcomes of radiotherapy in UM is limited because of missing data on radiation doses as well as great heterogeneity of study protocols. Radiation therapy outcomes are so far not comparable. Therefore, we recommend for upcoming studies on this topic to provide the biological effective dose (BED) or the equivalent dose in 2 Gy fractions (EQD_2_) per eye structure, thereby enabling a comparison of outcomes of different forms of radiation therapy.

## 1 Introduction

Posterior uveal melanoma (UM) is the most common primary intraocular tumor [1]. Making up 3–5% of all malignant melanomas, it is prone to metastasis, which significantly reduces life expectancy [2]. Incidence is estimated with about 5.1 cases per million per year worldwide [3].

Radiotherapy is first choice of treatment in small to medium sized primary UM and can be divided into brachytherapy and teletherapy. Brachytherapy is the preferred option for small to medium-sized tumors. The most commonly employed isotopes for UM are iodine-125 (I-125) and ruthenium-106 (Ru-106), the former being preferably used in the U.S.A., the latter in Europe [4]. Teletherapeutic radiation include proton therapy and stereotactic photon therapy using CyberKnife, Gamma Knife or the LINAC linear accelerator. Furthermore, adjuvant surgical therapies such as transscleral resection or endoresection can be used for large tumors to avoid toxic eye syndrome [5].

Studies comparing these radiotherapy options for UM are rare, mostly comparing a singular form of radiotherapy to an adjuvant therapy (e.g., TTT) or surgical procedure (e.g., enucleation or adjuvant tumor resection). Randomized controlled studies including two different forms of radiotherapy and comparing the outcomes have not been conducted so far.

Therefore, the objective of this systematic review and meta-analysis is to evaluate and compare different types of UM radiotherapy considering local tumor control, visual acuity, metastasis, survival, eye preservation and radiation side effects.

## 2 Material & methods

We carried out a systematic review and meta-analysis based on the Cochrane Handbook for Systematic Reviews [6].

### 2.1 Literature search and search criteria

This systematic review concentrates on choroidal and ciliary body melanomas, which are summarized as posterior uveal melanoma. Due to various different aspects regarding diagnosis and therapy, iris lesions are excluded. The MEDLINE database search was performed to identify studies of all languages investigating efficacy and side effects of radiotherapy in primary UM published from January 1st, 2000, to December 31st, 2021, and therefore also included Collaborative Ocular Melanoma Study (COMS) reports. Measured outcomes were local tumor control, overall survival, visual acuity, enucleation rate, radiation side effects and metastasis, based on radiation dose emitted to the tumor. RCTs, prospective cohort studies and retrospective cohort studies were the included study designs. Included interventions were all currently available forms of radiation therapy, e.g., proton therapy, CyberKnife, Gamma Knife, brachytherapy and (fractionated) radiotherapy (f)SRT. Studies including two study arms comparing different types of therapy have only been considered when the data for each radiation therapy have been able to be isolated and analyzed by itself. Only studies with adult patients (18 years of age or older), who have been diagnosed with UM for the first time and have undergone radiotherapy as first line therapy, were included in this review. Studies examining patients with pre-existing conditions such as diabetes mellitus or high blood pressure were also included. Studies on children below 18 years, on pregnant patients as well as on patients who have undergone therapy other than listed above, were excluded.

### 2.2 Study selection and data extraction

Overall, 4886 studies were manually screened in title and abstract for the eligible outcomes presented in Table 1a, Table 1b. A total of 406 potential studies were identified in the title-abstract-screening and further selected by reading the full text. The selection process was performed by 2 independent reviewers.

**Table 1a:** Characteristics of selected RCTs The table shows the characteristics of the selected RCTs eligible for further critical appraisal and statistical analysis.

Author	Country (year)	Study design	Type of radiotherapy	No. Patients	Patient characteristics male/female (%); age (mean)	Outcomes
Jampol et al.	USA (2002)	Randomized, controlled, clinical trial	I-125 brachytherapy vs. enucleation	638 (only brachytherapy)	m 50 %/f 50 %; 61 years (median)	Local tumor control, Enucleation
Nanda et al.	USA (2002)	Randomized, controlled, clinical trial	I-125 brachytherapy vs. enucleation	657 (only brachytherapy)	m 50 %/f 50 %; 61 years (median)	Visus

**Table 1b:** Characteristics of selected NRSI The table shows the characteristics of the selected pro- and retrospective NRSI eligible for further critical.

Author	Country (year)	Study design	Type of radiotherapy	No. Patients	Patient characteristics male/female (%); age (mean)	Outcomes
Prospective, non-randomized studies						
Sánchez-Tabernero et al.	Spain (2017)	Prospective, consecutive, interventional case series	I-125 brachytherapy	311	m 46 %/f 54 %; 59.2 years	Local tumor control
Polishchuk et al.	USA (2016)	Prospective cohort study	Proton beam radiation therapy	645	m 51 %/f 49 %; 60.3 years	Visus
Marinkovic et al.	Netherlands (2016)	Prospective case-control study	Ruthenium-106 brachytherapy (Comparison with Ru-106 + TTT)	253	m 46 %/f 54 %; 63.5 years	Local tumor control Survival Eye preservation Visus Metastasis
Lane et al.	USA (2015)	Prospective cohort study	Proton beam radiation therapy	3088	m 49.9 %/f 51.1 %; 61.3 years (median)	Survival
Mishra et al.	USA (2013)	Prospective cohort study	Proton beam radiation therapy	704	m 49.6 %/f 50.4 %; 60 years	Neovascular glaucoma
García-Álvarez et al.	Spain (2012)	Prospective cohort study	I-125 brachytherapy vs. enucleation	126	m 53 %/f 47 %; 58.3 years	Local tumor control Visus Eye preservation Survival
Muller et al.	Netherlands (2011)	Prospective cohort study	Fractionated stereotactic radiation therapy (fSRT)	102	m 57 %/f 43 %; 63 years (median)	Local tumor control Visus Radiation side effects Metastasis free survival
Mosci et al.	Italy (2008)	Prospective cohort study	Proton beam radiation therapy	368	m 49.5 %/f 50.5 %; 62.3 years	Local tumor control Eye preservation Survival
Muller et al.	Netherlands (2005)	Prospective cohort study	Fractionated stereotactic radiation therapy (fSRT)	38	m 55 %/f 45 %; 61 years	Local tumor control Visus Radiation side effects
Damato et al.	UK (2005)	Prospective cohort study	Ru-106 brachytherapy	458	m 43 %/f 57 %; 60.9 years	Visus
Damato et al.	UK (2005)	Prospective cohort study	Ru-106 brachytherapy	458	m 43 %/f 57 %; 60.9 years	Local tumor control
Höcht et al.	Germany (2004)	Prospective cohort study	Proton beam radiation therapy	245	m/f n.a.; 58.5 years	Local tumor control Eye preservation Radiation side effects
Mueller et al.	Germany (2003)	Prospective cohort study	Gamma knife	100	m 51 %/f 49 %; 62 years	Local tumor control Radiation side effects (Rubeosis iridis/secondary glaucoma) Enucleation Visus
Miguel et al.	Spain (2018)	Prospective cohort study	I-125 brachytherapy vs. enucleation	185	m 44 %/f 56 %; 61 years	Visus
Gragoudas et al.	USA (2002)	Prospective cohort study	Proton beam radiation therapy	2069	m 49 %/f 51 %; 61 years	Local tumor control Survival Visus Enucleation
**Retrospective, non -randomized studies**						
Dunavoelgyi et al.	Austria (2011)	Retrospective cohort study	Hypofractionated stereotactic radiation therapy (fSRT)	212	m 57 %/f 43 %; 60.0 years	Local tumor control Enucleation Metastasis Survival
Krohn et al.	Norway (2007)	Retrospective cohort study	I-125 Brachytherapy	108	m 49 %/f 59 %; 66 years (median)	Visus Eye preservation Radiation side effects Survival
Damato et al.	UK (2005)	Retrospective cohort study	Proton beam radiation therapy	349	m 54 %/f 46 %; 57.7 years	Local tumor control Visus Metastasis free survival

Extracted data included bibliographic data (author, year, title, study type, country, PMID), follow-up times, pre-conditions, data on outcomes (i.e., overall survival, recurrence, enucleation rate, visual acuity, metastasis), tumor data (size, location), radiation side effects and dose rates. Table 2a, Table 2b in the supplementary shows a detailed list of all 405 studies selected for full text screening.

**Table 2a:** Risk of bias assessment of non-randomized studies of intervention with the Cochrane ROBINS-I tool The table shows the results of the risk of bias assessment using the ROBINS-I tool of the Cochrane Collaboration. Overall, there are seven domains (D1 – D7) analyzed. All 18 studies were rated moderate [8] or serious [10]. No study received a low or critical rating.

Study	D1	D2	D3	D4	D5	D6	D7	Overall
Gragoudas et al. (2002)	Low	Low	Low	Low	Moderate	Moderate	Moderate	Moderate
Krohn et al. (2008)	Serious	Low	Serious	Low	Low	Moderate	Moderate	Serious
Sánchez-Tabernero et al. (2017)	Serious	Low	Low	Moderate	Low	Low	Moderate	Serious
Polishchuk et al. (2017)	Moderate	Low	Low	Low	Moderate	Low	Moderate	Moderate
Marinkovic et al. (2016)	Moderate	Low	Low	Low	Moderate	Moderate	Moderate	Moderate
Lane et al. (2015)	Serious	Low	Low	Low	Low	Low	Moderate	Serious
Mishra et al. (2013)	Low	Low	Low	Low	Low	Low	Moderate	Moderate
García-Álvarez et al. (2012)	Serious	Low	Low	Low	Low	Moderate	Moderate	Serious
Muller et al. (2012)	Serious	Low	Moderate	Low	Low	Moderate	Moderate	Serious
Dunavoelgyi et al. (2011)	Moderate	Low	Low	Low	Low	Low	Moderate	Moderate
Mosci et al. (2009)	Serious	Low	Low	Low	Moderate	Low	Moderate	Serious
Muller et al. (2005)	Moderate	Low	Low	Low	Moderate	Low	Moderate	Moderate
Damato et al. [2005a]	Serious	Low	Low	Low	Low	Low	Moderate	Serious
Damato et al. [2005b]	Low	Low	Low	Serious	Serious	Serious	Moderate	Serious
Damato et al. [2005c]	Low	Low	Low	Moderate	Moderate	Low	Moderate	Moderate
Höcht et al. - (2004)	Serious	Low	Serious	Moderate	Moderate	Low	Moderate	Serious
Mueller et al. (2003)	Serious	Low	Moderate	Low	Moderate	Low	Moderate	Serious
Miguel et al. (2018)	Low	Low	Low	Low	Low	Moderate	Moderate	Moderate

**Table 2b:** Risk of bias assessment of the RCTs with the Cochrane RoB 2 tool The table shows the results of the risk of bias rating with the RoB 2 tool of the Cochrane Collaboration. Overall, there are five domains (D1 – D5) analyzed. Both RCTs received an overall rating of „some concerns“. No study received the overall rating of "low".

Study	D1	D2	D3	D4	D5	Overall
Jampol et al. COMS No. 19	Some concerns	Low	Low	Low	Low	Some concern
Nanda et al. COMS No. 22	Some concerns	Low	Low	Low	Low	Some concern

### 2.3 Critical appraisal

The assessment of risk of bias (RoB) was carried out using the Cochrane Risk of Bias Tool. For non-randomized trials (NRSI), the ROBINS-I tool [7] was used in accordance with Chapter 25 of the Cochrane Collaboration Handbook [8]. For randomized controlled trials (RCTs), the ROB 2 tool [9] was used in accordance with the Cochrane Collaboration Handbook [10]. For each of the 20 studies selected, critical appraisal was performed using these two assessment tools. Assessment of risk of bias included low, moderate, serious, and critical judgement.

Analysis of publication bias by funnel plot requires enough studies (>10 studies). Therefore, we waived from performing this analysis [11].

### 2.4 Meta-analysis

Extracted data were used for meta-analysis. Out of the final 20 studies, 10 studies on I-125 brachytherapy and proton therapy were eligible for further analysis.

Heterogeneity analysis was performed to identify potential studies for meta-analysis. The study outcomes vary due to differences in study design and bias (methodological diversity) as well as study population, therapeutic differences between various countries and treatment centers, radiations doses or different outcome measurement tools (clinical diversity) [6]. Due to slight variances in radiation doses between the studies, the results should be accepted cautiously as the influence of these variances cannot be validly quantified at this point [12]. Due to the heterogeneity of the selected studies, the random effects model was chosen. The data obtained from the studies are converted into a uniform, meaningful measure, the effect measure, and presented by means of forest plots. To quantify heterogeneity, primarily tau^2^ and I^2^ were used. Tau^2^ quantifies the standard deviation of proportions. I^2^ represents the proportion of variance between studies and thus the variance of the true effect of the therapy [13]. Data synthesis and visualization was performed using R (R version 4.1.3, library: meta, procedure: metaprop).

## 3 Results

### 3.1 Search results and study selection

**Fig. 1:**
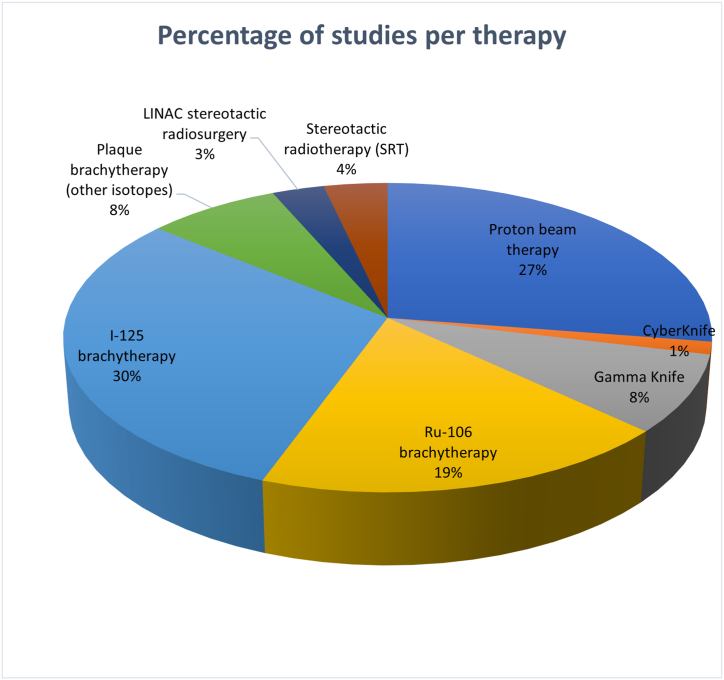
Proportion of selected studies with regards to type of radiotherapy This figure shows the proportion in percent of a total of 306 selected studies with regards to the radiotherapy which is analyzed.

We identified 4886 studies in MEDLINE. The title-abstract-screening showed a total of 406 studies eligible for full-text-screening. Among these, 306 studies exclusively examined radiotherapy. 100 studies examined, among other aspects, radiotherapy plus TTT or enucleation or palladium-103 or helium brachytherapy, which were excluded from the systematic review. The proportion of all included 306 studies is shown in Fig. 1.

**Fig. 2:**
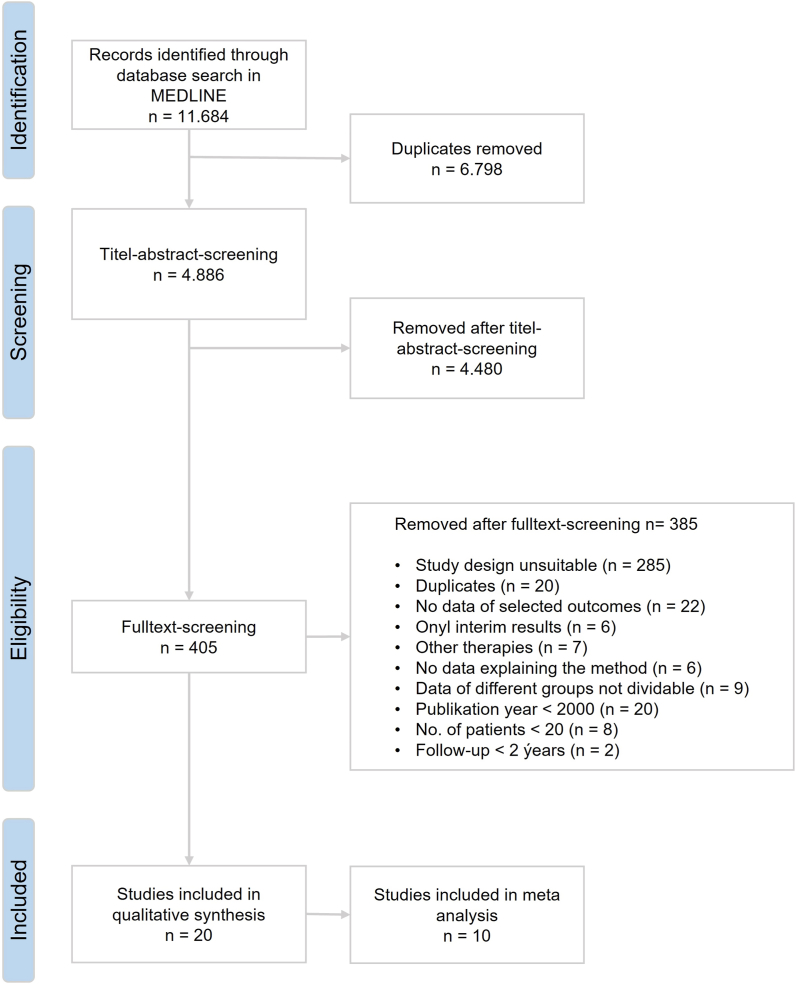
Systematic literature search flow diagram The figure shows the systematic literature search as flow diagram. A total of 20 studies are eligible for qualitative synthesis. Of these, 10 studies are eligible for meta-analysis.

After screening the titles, abstracts, and full texts, we identified 20 eligible studies which met the inclusion criteria (PICO). Publishing countries were the U.S.A. and European countries (UK, Spain, Netherlands, Germany, Norway, and Austria). Patients of included studies were treated with an included form of radiotherapy and at least 1 of the defined outcomes were analyzed. 9 of these studies investigated brachytherapy (I-125 and Ru-106), 7 are proton therapy studies, 3 are stereotactic radiotherapy studies and 1 study analyzed Gamma Knife. Two of these studies were RCTs, carried out by the COMS study group. All other 18 studies were divided into 15 prospective and 3 retrospective cohort studies. 14 studies followed a single-arm study design. Out of these 20 studies (2 RCTs + 18 others), 10 were eligible for meta-analysis. The selection process is shown in Fig. 2. Tables of study characteristics are shown in Table 1 a) and b) for RCTs and NRSI respectively.

### 3.2 Assessment of risk of bias

Risk of bias was assessed using the ROBINS-I and RoB 2 tools from the Cochrane Collaboration [7,9]. The ROBINS-I assessment was used for 18 non-randomized studies of intervention, resulting in 8 studies receiving a moderate risk of bias and 10 studies receiving a serious risk of bias. An overall assessment of low risk of bias would signify comparability with a well-performed RCT. Since most of the studies included were single-arm cohort studies (3 of them retrospective), they had not enough statistical significance to be comparable to a RCT.

The RoB 2 assessment was used for the 2 RCTs, both showing only some concerns regarding their risk of bias. Results are presented in Table 2 a) and b).

### 3.3 Results of meta-analysis

#### 3.3.1 Heterogeneity analysis

We selected 10 studies for our meta-analysis out of the 20 studies eligible for qualitative synthesis. We combined studies with the same type of radiotherapy plus analyzing the same outcomes. By this means, 8 useful therapy-outcome combinations were merged to perform heterogeneity analysis.

The heterogeneity analysis showed that heterogeneity of studies is low and sufficient for 3 out of 8 therapy-outcome combinations: local tumor control with I-125 brachytherapy, local tumor control with proton therapy and eye preservation with I-125 brachytherapy.

#### 3.3.2 Random Effects Model

Due to heterogeneity of the selected studies, the random effects model was chosen. The study data analyzed in the Random Effects Model is shown in Table 3. A total of 9 data sets (3 data sets for each therapy-outcome combination) is analyzed. The data obtained from these studies were converted into a uniform value, which is the effect measure, and presented via forest plot (Fig. 3a) – c)). The forest plot is a graphical representation of pooled study results frequently used in meta-analysis, which enables the reader to acquire large amount of information immediately. The small black squares next to the studies in Fig. 3 a) – c) are individual effect estimators, while the diamond represents the pooled results of all studies.

**Table 3:** Studies analyzed in the random effects model with RoB assessment The table shows a list of studies for meta-analysis using the random effects model as a basis for the creation of forest plots. For a better overview, the respective RoB rating for the studies has been added. This influences the interpretation of the results.

Study (country)	No. of patients (sex; age)	Study design	Outcome	Therapy	Cases/Total	RoB assessment
Jampol et al., 2002 (USA)	638 (m 325/f 325; 61 years (median))	RCT	Local tumor control	I-125 brachytherapy	581/638	
Sanchez-Tabernero et al., 2017 (Spain)	311 (m 144/f 167; 59.2 years (mean))	Prospective cohort study	Local tumor control	I-125 brachytherapy	295/311	
Garcia-alvarez et al., 2012 (Spain)	126 (m 53 %/f 47 %; 58.3 years (mean))	Prospective cohort study	Local tumor control	I-125 brachytherapy	132/136	
Gragoudas et al., 2002 (USA)	2069 (m 49 %/f 51 %; 61 years (median))	Prospective cohort study	Local tumor control	Proton therapy	2024/2069	
Mosci et al., 2009 (Italy)	368 (m 49.5 %/f 50.5 %; 62.3 years (mean))	Prospective cohort study	Local tumor control	Proton therapy	337/368	
Damato et al., 2005 (UK)	349 (188 m/161 f; 57.7 years (mean))	Prospective cohort study	Local tumor control	Proton therapy	340/349	
Jampol et al., 2002 (USA)	638 (m 325/f 325; 61 years (median))	RCT	Eye preservation	I-125 brachytherapy	569/638	
Krohn et al., 2007 (Norway)	108 (m 49/f 59; 66 years (median))	Retrospective cohort study	Eye preservation	I-125 brachytherapy	52/56	
Garcia-alvarez et al., 2012 (Spain)	126 (m 53 %/f 47 %; 58.3 years (mean))	Prospective cohort study	Eye preservation	I-125 brachytherapy	126/136

**Fig. 3a:**
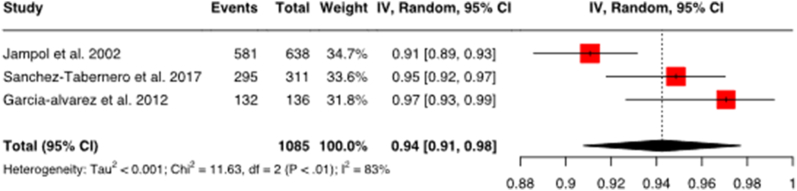
Forest plot for local tumor control with I-125 brachytherapy The figure shows the forest plot for local tumor control with I-125 brachytherapy. The combined effect size is 0.94 with a confidence interval of 0.91–0.98. This means that 94 % of patients on I-125 brachytherapy have achieved local tumor control. The confidence level is 95 %. Heterogeneity: Tau2 = <0.001; Chi2 = 11.63, df = 2 (P < .01); I2.

#### 3.3.3 Local tumor control with I-125 brachytherapy

For local tumor control with I-125 brachytherapy (Fig. 3a), studies comprising a total of 1085 patients were evaluated. The pooled proportion of patients with local tumor control on I-125 brachytherapy from the three studies is 0.94, meaning that 94 % of patients with I-125 brachytherapy have achieved local tumor control. The largest study with the highest weighting by Jampol et al. receives a rating of "some concerns" in the RoB assessment and is thus rated best among the three studies included.

#### 3.3.4 Local tumor control with proton therapy

For local tumor control with proton therapy (Fig. 3b), 3 studies comprising a total of 2786 patients were evaluated. The proportion of proton therapy patients with local tumor control, pooled from the three studies, is 0.96. This indicates that 96 % of patients with proton therapy have achieved local tumor control. However, the conclusion that proton therapy thus results in more effective tumor control than I-125 brachytherapy (with 94 % local tumor control) cannot be drawn, as too few studies were included in the analysis and a second study arm is missing to validate the results against a control group. Further well-performed randomized controlled trials would be necessary to verify the results.

**Fig. 3b:**
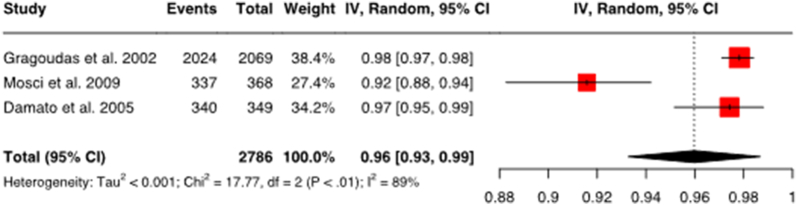
Forest plot for local tumor control with proton therapy The figure shows the forest plot for local tumor control with proton therapy. The combined effect size is 0.96 with a confidence interval of 0.93–0.99. This means that 96 % of patients on proton therapy have achieved local tumor control. The confidence level is 95 %. Heterogeneity: Tau2 = <0.001; Chi2 = 17.77, df = 2 (P < .01); I2: 89 %.

#### 3.3.5 Eye preservation with I-125 brachytherapy

For the outcome of eye preservation with I-125 brachytherapy (Fig. 3c), 3 studies with a total of 830 patients were evaluated. The pooled proportion of patients with eye preservation on I-125 brachytherapy from the three studies is 0.91. This signifies that 91 % of I-125 brachytherapy patients were able to retain their eye.

**Fig. 3c:**
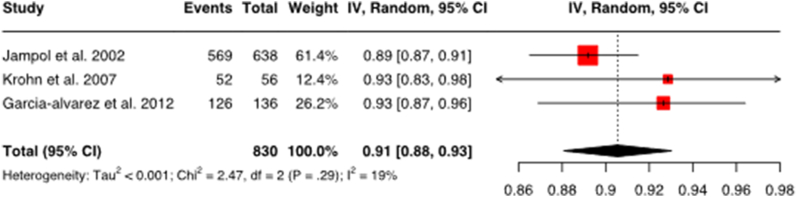
Forest plot for eye preservation with I-125 brachytherapy The figure shows the forest plot for eye preservation with I-125 brachytherapy. The combined effect size is 0.91 with a confidence interval of 0.88–0.93. This means that 91 % of patients with I-125 brachytherapy were able to retain their eye. The confidence level is 95 %. Heterogeneity: Tau2 = <0.001; Chi2 = 2.47, df = 2 (P = .29); I2: 19 %.

In this case, the study by Jampol et al. once more receives the highest weighting and a rating of "some concerns" in the RoB assessment, which is the best result among the three studies analyzed.

As the currently published studies for this rare disease comprise mostly single-arm analysis, the interpretation of our data needs to be handled with care. The pooled outcome estimates are the best available analysis we can achieve for our evidence compilation on therapy effects. Also missing data on radiation dose parameters that make two types of radiotherapies comparable to one another is a key factor for substantiated evidence-based analysis.

## 4 Discussion

Up to date, radiotherapy is considered as standard care for patients diagnosed with primary UM. Rates of local tumor control, eye preservation and overall survival are excellent and comparable to enucleation as primary therapy. In this analysis, we have systematically reviewed the effects and side effects of radiation therapy for primary posterior uveal melanoma. Moreover, we have performed meta-analysis and estimated high local tumor control rates for I-125 brachytherapy and for proton therapy, in addition to a high rate of eye preservation for I-125 brachytherapy. Despite the existence of numerous publications on this topic, most studies lack an adequate control group as well as a radiation dose protocol, which would facilitate a comparison of different forms of radiotherapy. With this systematic review, we have endeavored to fill this gap. We researched and evaluated studies on radiotherapy for primary UM which analyzed outcomes like local tumor control, overall survival, eye preservation, visual acuity, metastasis and radiation side effects. Through systematic literature research and critical appraisal with validated instruments from the Cochrane collaboration (ROBINS-I and RoB 2 tools), we were able to identify strengths and weaknesses of current studies in the field of UM radiotherapy. However, comparing and evaluating studies that report on these outcomes has proven to be a difficult task, considering the various interrelated parameters upon which such outcomes depend. Some of these parameters relate to the disease itself, such as tumor size, tumor location or measurement of visual acuity. Other parameters pertain to the form of therapy, such as technical equipment of different treatment centers. Furthermore, the study design (e.g., case selection, randomization) plays a crucial role in comparability of treatment effects [14]. Another cause of bias may lie in the fact that some treatments are generally employed for larger tumors while other treatments are more often used on smaller tumors. This circumstance then consequently effects dose parameters as well. For example, Iodine-125 brachytherapy is preferred to Ru-106 in larger UMs because I-125 plaques provide twice the depth of penetration as Ru-106 plaques [15]. Therefore, a direct comparison of I-125 with Ru-106 brachytherapy may be misleading.

### 4.1 Study outcomes

#### 4.1.1 Local tumor control

Local tumor control was evaluated in a total of 12 of the 20 selected studies, ranking from 89.3 % to 98 % in 5 years. Our meta-analysis showed a local tumor control of 94 % with I-125 brachytherapy and 96 % with proton therapy.

In a recent 2022 review, Bilmin et al. [16] assume a local tumor control of 94 % for radiotherapy in general, compared to 81 % for enucleation. These results coincide with our results and other studies [17,18], inferring that good local tumor control (>89 % in 5 years) can be achieved through radiotherapeutic measures.

#### 4.1.2 Eye preservation

Data on eye preservation showed a 91 % chance of retaining the eye with I-125 brachytherapy. Mosci et al. [20] presented an eye preservation rate of 88.3 % with proton therapy after 4 years whereas Höcht et al. [14] observed a rate of 87.5 % after 3 years. This data is comparable to other publications. However, the conclusion of I-125 brachytherapy being more effective than proton therapy is erroneous. Patient selection, study centers and follow-up protocols are not harmonized as would be the case in a well-performed RCT comparing both types of radiotherapy.

#### 4.1.3 Metastasis

The rate of metastasis was investigated in 4 of the studies we included [[21], [22], [23]]. Overall, metastasis-free survival after 5 years was consistently reported in all studies. Rates ranged from 75 % to 93.8 %. The highest rates (90 % and 93.8 %) were achieved with proton therapy and Ru-106 brachytherapy, respectively. Stereotactic irradiation, by contrast, resulted in lower rates. The data for proton therapy and fSRT (fractionated stereotactic radiation therapy) is comparable to other studies which treated large tumors [24,25]. In a retrospective study by Cohen et al., 196 patients were either irradiated with Gamma Knife (78 patients) or enucleated (118 patients). Multivariate analysis showed that the chosen procedure had no significant effect on the rate of metastasis, while tumor size and ciliary body involvement were each significantly related to metastasis-free survival. This corresponds with current guideline recommendations, in which poor prognosis for metastasis depends on TNM classification (i.e. higher tumor size) and recurrence after radiotherapy.

#### 4.1.4 Overall survival

Survival after completion of radiotherapy was investigated in a total of 6 studies. Of these, 3 studies used brachytherapy, 2 studies used proton therapy, and 1 study used fractional stereotactic radiotherapy (fSRT). The average follow-up time ranged from 3 to 13.4 years. Most of the studies report overall survival and UM-specific survival at about 5 years (4.6–6 years). Two studies [26,27] describe a peak in UM-related deaths in the first 3–6 years after treatment.

Overall survival ranged from 78.8 % to 98 %. UM-specific survival ranged from 86.6 % to 99 %. The highest and lowest values were provided by brachytherapy studies. A comparison with the literature shows that tumor size in particular is decisive for prognosis and thus survival [19].

#### 4.1.5 Visual acuity

Since preservation of the eye and its visual functions is of relevance to the patient, visual acuity was among the most frequently investigated endpoints. A total of 12 studies collected data on visual acuity. Among them, all forms of radiotherapy included by us were represented. As a uniform study protocol does not exist, the data mostly is not comparable. However, since the data of radiation doses is not comparable due to missing data on biological effective dose (BED) or equieffective radiation dose (EQD_2_) per eye structure, there would not be sufficient statistical evidence. Visual acuity before the start of therapy also plays a role in maintaining vision after radiation. For example, patients with retinal detachment or tumor-related macular degeneration have a worse baseline compared to asymptomatic patients [15,28]. The location of the tumor and the dose of radiation on the optic nerve also influence vision retention [29].

#### 4.1.6 Radiation side effects

Only 5 of the studies selected in this review, examined side effects of radiation. These included 3 stereotactic radiation studies, as well as 1 study with proton therapy and brachytherapy, respectively.

The studies on stereotactic radiotherapy consist of 2 studies by Muller et al. from the Netherlands (2005 and 2011) as well as one study by Mueller et al. from Germany (2003). In their 2005 study on 38 patients, Muller et al. reported that the most common late adverse reactions one year after therapy were neovascular glaucoma (5 %), radiation retinopathy (5 %), radiation opticopathy (9 %), dry eyes (9 %) and retinal hemorrhage (5 %) [30]. 5 % of patients developed a cataract. 6 years later, they published another study on fractional stereotactic radiotherapy with 102 patients [22]. In this study, 10 % of patients developed a grade 3 cataract. About 19 % of patients developed radiation retinopathy and about 13 % developed radiation optic neuropathy In the study by Mueller et al., the proportion of patients with rubeosis iridis followed by secondary glaucoma was 12 % and 15 % after 1 and 2 years, respectively [31].

The results are comparable to other studies on stereotactic radiotherapy. In their study of 189 patients with tumor thickness of up to 12 mm, Van Beek et al. [32] reported cataract as the most common side effect (67.8 %), followed by radiation retinopathy (35.1 %), radiation maculopathy (23.8 %), vitreous hemorrhage (20.1 %), neovascular glaucoma (NVG) (20.0 %) and radiation opticopathy (12.4 %). Patients with anterior uveal melanoma were more likely to develop cataracts (p = .047, multivariable analysis).

### 4.2 Methodological aspects and limitations

The synthesis of scientific evidence includes critical evaluation of the selected studies, thereby identifying overestimated effects due to methodological weaknesses in the studies. This includes risks of bias, inconsistency (clinical or statistical heterogeneity) or imprecision, which may result in Type I or Type II errors [33]. Sources of potential bias in systematic reviews are, for instance, bias due to confounding, selection bias or bias in measurement of the outcomes. For non-randomized studies, there are only few validated instruments for assessing study quality. Especially in our collection of predominantly one-arm studies without a control group, the significance of the study results must be treated with reservations. A validated tool for the evaluation of non-randomized trials is the Risk of Bias Assessment tool ROBINS-I of the Cochrane Collaboration [7]. Due to the predominant single-arm study design without a control group, which were selected for this systematic review, the quality of the evidence was not assessed using the GRADE system [34].

The heterogeneity between the studies in this review was caused by varying study designs, methods for recruiting patients and measurement methods employed by study centers in different countries (U.S.A. and Europe). Furthermore, publication bias cannot be ruled out.

The specification of the *Biologically Effective Dose (BED)* or the Equivalent Dose *(EQD)* was not published in any of our analyzed studies except for one [35]. These are important values with which the dose per irradiated tissue type is standardized, thereby enabling a comparison between different types of radiation therapy. The aim of EQD is to be able to compare different forms of irradiation (in terms of dose or fractionation) with regard to the effect they achieve in the tissue [36]. A common clinical approach to this topic is the use of equivalent dose in 2-Gy fractions (EQD_2_) [36]. However, therapy options such es external beam radiation are very different in terms of physics, compared to plaque brachytherapy, for example. A direct comparison of radiation therapies using BED or EQD is difficult and should always include radiologists’ expertise to take into account the differing physical aspects between the radiation therapies. Our review shows the limitations of a direct comparison between the different therapy options and the reporting of BED or EDQ can only be an attempt to compare therapies by approximation.

As an additional methodological aspect, we would like to stress that the available data was extracted directly from the publications. For future studies, a pooled reanalysis, that is a quantitative merging of the original data, may be conducted. For this purpose, the original study data (individual data, i.e., all information on age, gender, or diagnosis available at the level of the individual) is required. To go even further, a meta-analysis may also be planned prospectively by creating a joint study protocol for the individual studies in advance [37].

**Fig. 4:**
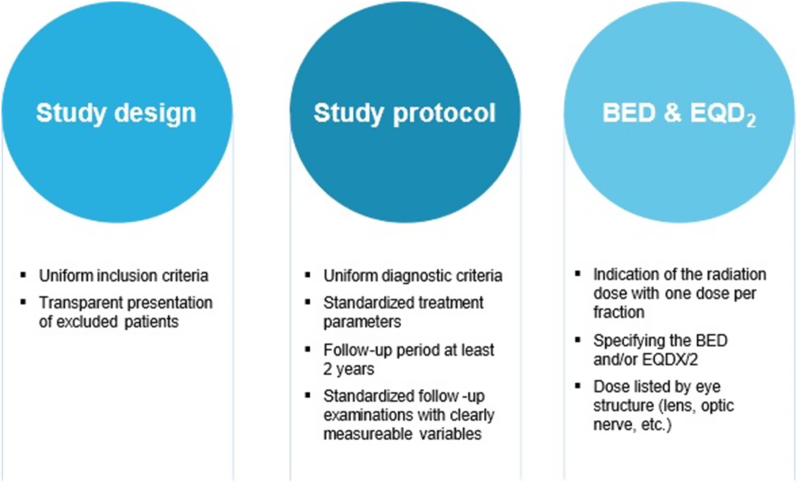
Recommendations for future studies This figure shows an overview of the recommendations which have been developed in this systematic review and which should be used for future studies to achieve comparability of radiation treatments.

The overview of current studies shows that all types of radiotherapy seem to cause similar effects and side effects. In addition to radiation dose and tumor size (which proportionally affects the dose), factors promoting the development of radiation side effects are proximity to the optic nerve and thus sensitive structures of the eye. In this context, we recommend reporting details on dose to apex, scleral dose and dose on critical eye structures such as optic nerve, if applicable. In the future, comparative studies with tumor size and localization as well as comparable radiation doses for the tumor and healthy eye structures would be necessary to make more accurate predictions regarding the development of radiation damage. Our recommendations have been summarized in Fig. 4.

## Funding

Vira Labunska was supported by a scholarship from the 10.13039/501100004168University of Lübeck within the framework of the emergency program for the support for refugee scientists from Ukraine.

## Registration

The study protocol was registered at the International Prospective Register of Systematic Reviews (PROSPERO) under the ID: CRD42022311758.

## Data availability statement for this work

Not applicable for systematic reviews.

## CRediT authorship contribution statement

**K. Erikson:** Conceptualization, Data curation, Formal analysis, Investigation, Methodology, Resources, Software, Supervision, Validation, Visualization, Writing – original draft. **A. Heidenreich:** Data curation, Formal analysis, Methodology, Software, Supervision, Writing – review & editing. **V. Labunska:** Formal analysis, Methodology, Validation, Visualization, Writing – review & editing. **R. Beach:** Formal analysis, Methodology, Software, Validation, Visualization, Writing – review & editing, Data curation. **F. Cremers:** Methodology, Software, Supervision, Validation, Visualization, Writing – review & editing, Formal analysis. **D. Rades:** Methodology, Software, Validation, Visualization, Writing – review & editing, Formal analysis. **S. Grisanti:** Data curation, Investigation, Methodology, Project administration, Resources, Software, Validation, Writing – review & editing. **A. Katalinic:** Data curation, Supervision, Validation, Visualization, Writing – review & editing, Conceptualization, Formal analysis, Investigation, Methodology, Project administration, Resources, Software. **V. Kakkassery:** Conceptualization, Data curation, Formal analysis, Funding acquisition, Investigation, Methodology, Project administration, Resources, Visualization, Writing – original draft, Software, Supervision, Validation.

## Declaration of competing interest

The authors declare that they have no known competing financial interests or personal relationships that could have appeared to influence the work reported in this paper.
